# The Dynamics of Team Learning: Harmony and Rhythm in Teamwork Arrangements for Innovation

**DOI:** 10.1177/00018392231166635

**Published:** 2023-04-08

**Authors:** Jean-François Harvey, Johnathan R. Cromwell, Kevin J. Johnson, Amy C. Edmondson

**Affiliations:** 1 HEC Montréal; 2 University of San Francisco; 3 Harvard Business School

**Keywords:** teams, learning, innovation, team dynamics, ambidexterity

## Abstract

Innovation teams must navigate inherent tensions between different learning activities to produce high levels of performance. Yet, we know little about how teams combine these activities—notably reflexive, experimental, vicarious, and contextual learning—most effectively over time. In this article, we integrate research on teamwork episodes with insights from music theory to develop a new theoretical perspective on team dynamics, which explains how team activities can produce harmony, dissonance, or rhythm in teamwork arrangements that lead to either positive or negative effects on overall performance. We first tested our theory in a field study using longitudinal data from 102 innovation teams at a Fortune Global 500 company; then, we replicated and elaborated our theory in a study of 61 MBA project teams at an elite North American university. Results show that some learning activities can occur within the same teamwork episode to have harmonious positive effects on team performance, while other activities combine to have dissonant negative effects when occurring in the same episode. We argue that dissonant activities must be spread across teamwork episodes to help teams achieve a positive rhythm of team learning over time. Our findings contribute to theory on team dynamics, team learning, and ambidexterity.

Team learning is vital for innovation teams to achieve high levels of performance (Argote, 1999; Edmondson, 1999; Alexander and van Knippenberg, 2014; Edmondson and Harvey, 2018), but it remains a complex and variegated construct. Multiple types of learning activities—notably reflexive, experimental, contextual, and vicarious—have been identified as important for team innovation (Ancona and Caldwell, 1992a; Edmondson, 2002; Harvey et al., 2022). However, sometimes these activities mutually reinforce one another to improve performance (Bresman, 2010; Kostopoulos, Bozionelos, and Syrigos, 2015), while other times they mutually hinder one another to undermine performance (Choi, 2002; Wong, 2004; Marrone, 2010). Therefore, although each learning activity on its own has the potential to enhance innovation in teams (Edmondson, Dillon, and Roloff, 2007; Harvey et al., 2022), it remains unclear how learning activities can be combined most effectively over the course of an innovation project to produce overall positive effects on team performance (Kozlowski and Bell, 2008; Argote, Lee, and Park, 2020).

Theory on team dynamics is essential to addressing this topic because it explains how teams integrate various activities over time in a coordinated fashion to achieve desired goals. The most prominent theory, which builds upon the classic input-process-output (IPO) model of team behavior (McGrath, 1964; Hackman and Morris, 1975), argues that teams can integrate different activities over time through a series of teamwork episodes that link together in iterative cause-and-effect relationships (Marks, Mathieu, and Zaccaro, 2001; see also Ilgen et al., 2005). An episode captures a single IPO cycle that involves multiple activities directed at accomplishing a short-term goal, and multiple episodes are strung together over time to reach longer-term goals. This theory provides a valuable perspective to understand the dynamics of team learning; however, when applied to innovation teams, it faces limitations. Innovation often requires teams to engage in activities that have conflicting short-term goals, such as exploration and exploitation (March, 1991; Edmondson, 2002), and longer-term goals often change during a project as new possibilities emerge (Cromwell, Amabile, and Harvey, 2018). Therefore, teams pursuing innovation must be adept at coordinating different learning activities over time, but existing theory provides little guidance to understand how learning activities should occur within and across episodes.

To develop a richer and more complete theory on the dynamics of team learning, we considered which domains might investigate combinations of harmonious and dissonant elements over time, finding this to be a central question in music theory (Albert and Bell, 2002).^1^ According to Albert and Bell (2002: 586), music theory provides a valuable conceptual toolkit for understanding dynamic temporal processes in organizations, because “we are time-experiencing organisms: we act when we do because of the way we conceive of our lives in time. . . . Music theory helps uncover these patterns.” We argue further that music theory is particularly well suited to help resolve theoretical issues identified in explaining team learning and innovation, because it focuses on classifying core components of a larger temporal process and treating them as isolated entities to be integrated in various arrangements over time (Meyer, 1989; White, 1994). This idea is analogous to what research on team learning has done by identifying different types of learning activities that theoretically can be combined in ongoing, iterative processes during a project (Edmondson, Dillon, and Roloff, 2007; Bell, Kozlowski, and Blawath, 2012). However, where music theory is more advanced—and becomes useful for our objectives—is in the analysis of how isolated parts relate to each other in elegant and aesthetic combinations to achieve desired results. Accordingly, such theory has helped scholars develop a clear understanding of how varying isolated parts can fit together in highly complex yet effective arrangements over time (Meyer, 1989; White, 1994).

We find that three concepts in music theory can enhance our understanding of team learning dynamics for innovation. The first is *tonality*, which refers to a central note around which all other notes are built; departures from this note indicate a rise in tension, and returns to this note indicate a resolution of tension. The second is *harmony*, in which multiple notes can be played simultaneously to produce either harmonic or dissonant sounds in a musical arrangement. Finally, *rhythm* arises when one note is complemented by several other notes in a musical arrangement, which can simultaneously be in tension with each other and create a sense of stability and predictability when played in a repetitive sequence. By theorizing how disparate aesthetic elements work together to create a cohesive whole, organizational scholars can gain new insights on how different activities can be integrated to achieve a long-term goal in teams. Therefore, given prior interest in the effective combination of different types of team learning for innovation, our cross-disciplinary theorizing can produce valid explanations of how different learning activities can and should unfold over time.

When we apply music theory to team learning and innovation, each type of learning can be viewed as a different note, and various combinations of learning activities over time can produce different teamwork arrangements that yield better or worse outcomes. Our primary research question is, thus, how does the arrangement of different learning activities over time influence innovation teams’ performance? To address this question, we theorize that reflexive learning serves as the tonal activity of a teamwork arrangement, and other types of learning—experimental, contextual, and vicarious—serve as complementary activities. When these activities help teams pursue a congruent short-term goal as reflexive learning (i.e., exploitation) and occur in the same teamwork episode, the activities can produce harmonious positive effects on performance. But when the activities focus on a conflicting goal (i.e., exploration) and occur in the same teamwork episode, they combine to produce dissonant negative effects on performance. When these activities are instead separated across teamwork episodes, teams can achieve a positive rhythm of learning over time to improve overall performance. This effect occurs because reflexive learning facilitates a rise and resolution of tension over time that allows multiple individuals to have stronger shared understanding of their work.

We collected data from two research settings to test and validate this theory. First, we collected survey data at multiple points in time from 102 teams participating in an internal innovation contest held in a Fortune Global 500 company. Results showed that different team learning arrangements indeed had different effects on performance, consistent with our theory. When multiple learning activities occurred in the same teamwork episode, they created harmonious positive effects on performance when they focused on a congruent short-term goal (i.e., exploitation) and created dissonant negative effects when they pursued conflicting goals (i.e., exploration and exploitation). When these activities with conflicting short-term goals were spread across multiple teamwork episodes, however, the result was a positive rhythm of team learning that improved overall performance. To further understand the mechanism driving these effects, we conducted a follow-up study with 61 MBA teams that completed a class innovation project. Results not only provided additional support for our theory but also confirmed that reflexive learning serves as the tonal activity of a teamwork arrangement for innovation, and that greater coordination quality plays a crucial role in promoting a more positive rhythm of team learning over time.

This research makes several theoretical contributions. First, we develop a new perspective on team dynamics by integrating insights from music theory (Albert and Bell, 2002) to shed light on how teams can integrate various activities over time into effective teamwork arrangements that lead to overall better performance. Existing theory on team dynamics emphasizes the sequential nature of activities that can be linked through iterative cause-and-effect relationships (Marks, Mathieu, and Zaccaro, 2001; Ilgen et al., 2005). Although this model helps define the structure of team activities during a project, it does not provide guidance on how specific activities should be combined within and across episodes. In this study, we show that some activities are harmonious with each other and can improve performance when they occur in the same teamwork episode, while others are dissonant and should occur across teamwork episodes to improve performance. This theory helps resolve a longstanding puzzle on how to integrate various team learning activities to achieve success in innovation projects, and it may help resolve other puzzles in team dynamics more generally (McGrath, 1991; Weingart, 1997; Cronin, Weingart, and Todorova, 2011), especially when teams must coordinate multiple activities that have conflicting short-term goals.

Our findings also contribute to theory on team learning more specifically. At this point, research has largely theorized about team learning, with a static view of process (Edmondson, Dillon, and Roloff, 2007; Harvey et al., 2022), such that multiple learning activities are theorized to occur essentially in one episode that transforms a single set of inputs into a set of outputs (McGrath, 1964; Hackman and Morris, 1975). This is problematic because team learning does not consist of only one activity at one point in time; it includes various activities with different short-term goals, occurring both inside and outside teams (Ancona and Caldwell, 1992a; Edmondson, 2002; Harvey et al., 2022), and these activities must be integrated across time to achieve success (Kozlowski and Bell, 2008; Argote, Lee, and Park, 2020). Therefore, when scholars have applied a static view to these activities, some have found that team learning activities combine to harm performance (e.g., Wong, 2004), while others have shown they combine to enable performance (e.g., Bresman, 2010). A more dynamic view shows that teams can iteratively transition between different types of learning over time, which resolves the contradiction found in prior literature and charts new paths for future research to investigate.

Finally, we contribute to theory on ambidexterity by examining the tension between exploration and exploitation at the team level of analysis (Gupta, Smith, and Shalley, 2006; Peretti and Negro, 2006; Alexander and van Knippenberg, 2014). Edmondson (2002) argued that team-based structures enable *organizations* to balance exploration and exploitation by having different teams engage in different kinds of learning, such that new product development teams focus on exploration, whereas management or sales teams focus on exploitation. Other researchers have suggested an additive perspective on exploration and exploitation in teams (e.g., Gibson and Birkinshaw, 2004; Jansen et al., 2016), arguing that teams engage in both activities to achieve more-innovative results, but this overlooks the inherent tension between them. Our study introduces a new approach to ambidexterity, suggesting that teams may be able to manage the tension between exploration and exploitation by iterating between them across teamwork episodes to create a more stable and predictable rhythm of team learning over time.

## Theory and Hypotheses

Research has identified different types of team learning that have been aggregated into a taxonomy based on two underlying dimensions (Harvey et al., 2022). The first dimension refers to the *orientation* of team learning, which includes either exploration or exploitation (Edmondson, 2002; Taylor and Greve, 2006; Argote, Lee, and Park, 2020), and the second refers to the *locus* of team learning, occurring either inside or outside teams (Choi, 2002; Marrone, 2010; Wiese et al., 2022). Exploration involves searching for new ideas, strategies, or knowledge that can help teams identify alternative courses of action to accomplish goals. When it occurs inside a team, it is called *experimental learning*, which involves brainstorming, building prototypes, drawing sketches, and running tests to help teams discover new ideas that lead to more-creative solutions to problems (Lee et al., 2004; Gilson et al., 2005; Vera and Crossan, 2005; Harvey and Kudesia, 2023). When exploration occurs outside a team, it is called *contextual learning*, which involves gathering information from the environment to learn about competitors, discover technology trends, or identify customer needs that stimulate new insights for a project (Ancona and Caldwell, 1992b; Hansen, 1999; Keller, 2001; Wong, 2004; Harvey, 2023).

By contrast, exploitation involves processing information to evaluate ideas, develop clear strategies, and generally improve the efficiency by which teams accomplish work. When this occurs inside teams, it is called *reflexive learning* and involves considering different members’ perspectives on current or changing conditions, evaluating ideas, and explicitly discussing ways to improve processes and strategies (West, 1996; Edmondson, 1999). Reflexive learning is particularly valuable for innovation because it allows teams to build stronger mental models of task strategies and team capabilities (Mathieu and Rapp, 2009; Ren and Argote, 2011; Schippers, Edmondson, and West, 2014; Leblanc, Rousseau, and Harvey, 2022), which improves their ability to adapt to changing conditions and reach long-term goals (Gibson and Vermeulen, 2003; De Dreu, 2007; Byron et al., 2022). Exploitation outside of teams is called *vicarious learning* and involves drawing on others’ experiences to learn about key aspects of a project (Argote and Ingram, 2000; Bresman, 2010; Myers, 2022). For instance, teams can learn what has worked particularly well or not in the past, which can help them skip unnecessary steps and avoid reinventing the wheel (Edmondson et al., 2003; Tucker, Nembhard, and Edmondson, 2007).

Scholars generally agree that each learning activity is important for team innovation, but they disagree on whether or how to combine them (e.g., Wong, 2004; Bresman, 2010). One potential explanation for this conflict is that scholars have primarily held a static view of process in their studies of team learning (McGrath, Arrow, and Berdahl, 2000), relying on the classic IPO model of team behavior (McGrath, 1964; Hackman and Morris, 1975). This model theorizes that various team inputs (e.g., diverse backgrounds) have a direct influence on team processes (e.g., learning), which subsequently affect performance (e.g., innovation). Accordingly, this model suggests that all types of team learning occur at relatively the same time—that is, during the process part of the IPO model—leading to different combinations that either improve or hinder performance.

Taking a more dynamic view of team learning is essential to resolve this contradiction (Weingart, 1997; Cronin, Weingart, and Todorova, 2011). One of the most prominent models of team dynamics is the theory of teamwork episodes proposed by Marks, Mathieu, and Zaccaro (2001), which draws upon earlier work on goal setting (Locke and Latham, 1990). According to Marks, Mathieu, and Zaccaro (2001: 359), “[Team] performance trajectories most commonly consist of several I-P-O-type cycles that run sequentially and simultaneously.” Therefore, one IPO cycle represents an episode, in which teams engage in various activities to accomplish a short-term goal, and episodes can be strung together to reach longer-term goals of a project (see also Ilgen et al., 2005). An important feature of this theory is that each episode is goal-directed and can be demarcated over time, such that the conclusion of one episode typically marks the beginning of another. Although this model provides an elegant extension of the IPO model to capture team dynamics, it lacks more-specific guidance on which activities should occur “sequentially and simultaneously” during a project (Marks, Mathieu, and Zaccaro, 2001: 359). In short, we still know little about how various bundles of team activities fit together over time (McGrath, 1991; see also Weingart, 1997; Cronin, Weingart, and Todorova, 2011).

To elaborate theory on teamwork episodes, we draw on insights from music theory (Albert and Bell, 2002), which argues that aesthetic properties of music can be applied to organizational phenomena to help scholars better understand the timing of events. We use three concepts in particular—tonality, harmony versus dissonance, and rhythm—to develop new theory that explains how innovation teams can combine different learning activities over time to improve overall performance. These three concepts provide a foundation for future scholars to integrate additional insights from music theory into the team dynamics literature.

### Identifying the Tonal Activity of a Teamwork Arrangement

One of the most important characteristics of a musical arrangement is tonality, which provides a map of how notes, pitches, and chords should be arranged to produce a feeling of stability and direction (Ammer, 1972; Meyer, 1989; Albert and Bell, 2002). Within this structure, the note with the greatest stability is referred to as the tonal note, and all other notes are interpreted against it throughout the arrangement. For example, beginning a song with the tonal note establishes a baseline of what to expect, and when subsequent notes deviate from the tonal note, people perceive a rise in tension that gives the arrangement a feeling of movement and direction. The more these notes deviate from the tonal note, the greater the perceived tension, which then gets resolved when the music returns to the tonal note. This phenomenon has been described as the “law of return,” which states that “other things being equal, it is better to return to any starting point whatsoever than not to return” (Meyer, 1956: 151). Therefore, the tonality provides clear instructions on which specific notes can be played to create feelings of stability, tension, and directionality in a musical arrangement.

To apply the concept of tonality to team learning, we must determine which type of learning is most likely to serve as the tonal activity of a teamwork arrangement, against which all other learning activities are interpreted. According to music theory, the tonal note is the most stable; thus, the tonal activity must be sustainable during a project, meaning it can be repeated multiple times without undermining team processes and performance. With this view in mind, learning activities that focus on exploration can be ruled out because they emphasize divergence, novelty, and the search for new strategies to accomplish goals. Although these activities are essential for innovation (Edmondson, 2002; Taylor and Greve, 2006; Alexander and van Knippenberg, 2014; Argote, Lee, and Park, 2020; Harvey et al., 2022), they cannot be repeated sustainably during a project. For example, if teams repeatedly engage in experimental learning, they will continue to brainstorm new ideas and run tests to generate new insights for a project; and if they repeatedly engage in contextual learning, they will perpetually search for new information outside the team. In both cases, the team will fail to consolidate new ideas, strategies, and knowledge into a coherent plan to reach longer-term goals.

By contrast, learning activities focused on exploitation can potentially serve as the tonal activity because they emphasize convergence, efficiency, and consolidation of strategies to achieve goals. But the degree to which exploitation is sustainable depends on the locus of team learning. If teams repeatedly engage in vicarious learning, they will constantly gather insights external to the team on ways to improve the project. Although this may provide additional insights that improve team efficiency (Bresman, 2013), it can also limit their ability to consolidate external views and develop shared understanding of their own project goals and strategies (Miner et al., 1999). In contrast, reflexive learning provides teams with the tools needed to constantly iterate, update, and improve strategies over time to reach long-term goals (Vashdi et al., 2007; Bell, Kozlowski, and Blawath, 2012). Teams that repeat reflexive learning throughout a project can ensure they are incorporating diverse perspectives, modifying processes, and adapting strategies to develop a coherent plan for success (Swift and West, 1998; Schippers, Edmondson, and West, 2014; Konradt et al., 2021). Therefore, reflexive learning can reliably sustain itself over time, making it the most viable candidate to serve as the tonal activity of a teamwork arrangement for innovation.

Although reflexive learning may play a central role in helping innovation teams successfully complete a project, it is insufficient on its own to improve overall performance. Unlike projects that emphasize quality, efficiency, or accuracy, innovation teams must also develop outcomes that are novel or unique (West, 1990; Edmondson and Nembhard, 2009; Alexander and van Knippenberg, 2014; Anderson, Potočnik, and Zhou, 2014). Therefore, exploration is almost always necessary in teams to achieve more-innovative outcomes (March, 1991; Edmondson, 2002; Taylor and Greve, 2006; Alexander and van Knippenberg, 2014). Theory on teamwork episodes suggests that numerous possible combinations of team learning can occur within and across episodes so teams can achieve high levels of both exploration and exploitation. To investigate how such combinations should be arranged to achieve the best overall performance, we draw on additional principles of music theory.

### Harmony Versus Dissonance Within Teamwork Episodes

Recall that Marks, Mathieu, and Zaccaro (2001) have viewed teamwork episodes as a series of IPO processes strung together over time in cause-and-effect relationships. Within each episode, teams can engage in multiple activities simultaneously to achieve a short-term goal. Given that reflexive learning is the tonal activity of a teamwork arrangement, all other learning activities are interpreted against it. Therefore, we theorize that vicarious learning can operate in harmony with reflexive learning because both pursue a congruent short-term goal—exploitation. Reflexive learning can help team members share knowledge and develop a better understanding of the team’s capabilities, tasks, and strategies (West, 1996; Edmondson, 1999). Vicarious learning can enhance this understanding (Bresman, 2010; Bresman and Zellmer-Bruhn, 2013), because learning from experienced others can transfer additional knowledge to the team, raising awareness about other techniques and processes that can improve existing strategies (Epple, Argote, and Devadas, 1991; Darr, Argote, and Epple, 1995). When these two learning activities are combined, external knowledge can be easily integrated with internal knowledge to facilitate more-efficient progress toward a common goal (Hackman and Morris, 1975; Tucker, Nembhard, and Edmondson, 2007). Thus, we hypothesize the following:

**Hypothesis 1 (H1):** When reflexive learning and other harmonious learning activities occur in the same teamwork episode, they will combine to create a positive effect on innovation project performance.

By contrast, we argue that experimental and contextual learning are dissonant with reflexive learning because they focus on a different short-term goal—exploration. Although several studies have shown that innovation teams typically need to engage in both exploration and exploitation during a project (Taylor and Greve, 2006; Hülsheger, Anderson, and Salgado, 2009), they have not provided clear guidance on the timing of these activities. Because reflexive learning promotes the convergence of ideas to deepen knowledge and improve efficiency, while experimental and contextual learning promote the divergence of ideas to broaden knowledge and increase novelty (March, 1991; Edmondson, 2002; Harvey et al., 2022), combining them in the same teamwork episode can produce conflict that undermines a team’s ability to work together and integrate knowledge into a coherent strategy (Wong, 2004; Reagans, Miron-Spektor, and Argote, 2016). Such conflict can also reduce goal commitment from team members (Weldon and Weingart, 1993), negatively affecting the overall quality of team learning and undermining performance. Therefore, we hypothesize the following:

**Hypothesis 2 (H2):** When reflexive learning and other dissonant learning activities occur in the same teamwork episode, they will combine to create a negative effect on innovation project performance.

### Achieving Rhythm Across Teamwork Episodes

We argue that teams can combine dissonant activities such as exploration and exploitation most effectively by separating them across teamwork episodes. According to Marks, Mathieu, and Zaccaro (2001), the knowledge produced from one episode directly influences the knowledge produced in a subsequent episode, such that teams will accrue gains or losses over time that affect their overall performance at the end of a project. Similarly, in music, rhythm is defined as the interrelationship between different notes, such that “each has a beginning, an intent, and a final consummation and that the last stage of one gives rise to another” (Albert and Bell, 2002: 578). The tonal note can begin a musical arrangement to set expectations for subsequent notes; when these notes are dissonant with the tonal note, tension rises to evoke a feeling of unrest that causes people to instinctively anticipate a return to the tonal note (Meyer, 1989; Swain, 2002). Analogously, a teamwork arrangement can begin with the tonal activity of reflexive learning, which can be followed by other dissonant learning activities to create a similar rise in tension that must be resolved. Specifically, an initial episode of reflexive learning allows teams to take stock of available knowledge and skills, discuss how to assign responsibilities, and develop a strategy to reach long-term goals (West, 1996; Edmondson and Harvey, 2017). Furthermore, it can highlight knowledge gaps that need to be filled to resolve the uncertainty about long-term goals or strategies that is inherent to all innovation projects (March, 1991; Edmondson, 2002; Harvey et al., 2022).

After an initial episode of reflexive learning, teams must take action to solve the problem at hand, which is most likely to occur through learning activities involving exploration. With experimental learning, team members brainstorm new ideas and run tests to identify solutions that have the most potential (Lee et al., 2004; Harvey and Kudesia, 2023); and with contextual learning, teams collect new information from outside the team to learn about competitors or identify emerging trends in the field (Ancona and Caldwell, 1992b; Wong, 2004; Harvey, 2023). Vicarious learning can also be helpful at this stage because it provides additional insights about project strategies that teams may not have considered before (Szulanski, 2000; Edmondson et al., 2003; Bresman, 2013). Regardless of the activity, teams will experience some kind of tension compared to the initial expectations set in the first episode of reflexive learning. More-dissonant activities (i.e., experimental and contextual learning) will push teams to explore alternative courses of action and more-divergent strategies to achieve goals, while more-harmonious activities (i.e., vicarious learning) will help teams fine-tune existing strategies based on additional or complementary knowledge. Afterward, in line with the “law of return” (Meyer, 1956: 151, 1989), teams will seek to resolve this tension by engaging in a final episode of reflexive learning, allowing them to share ideas, evaluate possibilities, and synthesize knowledge into a new shared mental model for the project. Accordingly, we hypothesize the following:

**Hypothesis 3 (H3):** Two separate episodes of reflexive learning will be mediated by other types of team learning to create a positive rhythm of learning activities over time.

Yet, all sequences of team learning are not equal. According to music theory, the process of creating and resolving tension strongly influences the emotional impact and overall quality of a musical arrangement (Meyer, 1989). The greater the tension created and resolved, the more pleasing the overall experience. When we apply this principle to team learning, it suggests that some rhythms of learning activities may be more effective than others because they create and resolve a greater level of tension over time. Accordingly, we argue that activities involving exploration are likely to produce the strongest positive rhythm of learning in teams. Exploration is essential for innovation because it helps teams search for new ideas, knowledge, and strategies (Taylor and Greve, 2006; Alexander and van Knippenberg, 2014; Harvey et al., 2022), but it can also introduce tension by disrupting existing plans set forth through exploitation (March, 1991; Edmondson, 2002). When teams engage in these dissonant activities in the same teamwork episode, high levels of tension can lead to poor performance (e.g., Wong, 2004). But when these activities are separated across teamwork episodes, the result can be a rise and resolution of tension over time that provides a feeling of stability and direction in a project (Albert and Bell, 2002). And so, instead of pursuing exploration and exploitation simultaneously (e.g., Jansen et al., 2016), teams can dynamically iterate between them over time to create a positive rhythm of activities that promotes overall performance.

By contrast, a rhythm of team learning that involves more-harmonious activities—such as two episodes of reflexive learning mediated by vicarious learning—may not produce the same performance benefits on innovation projects. Although vicarious learning can help teams identify additional insights they did not consider before (Harvey et al., 2022), these insights are likely to focus on improving the efficiency and predictability of existing task strategies (Bresman, 2010), limiting the search for more-creative strategies to accomplish goals. As a result, teams are unlikely to discover unexpected breakthrough solutions that can vastly improve project performance (Amabile and Pratt, 2016; Edmondson and Harvey, 2017; Cromwell, Amabile, and Harvey, 2018). Therefore, vicarious learning does not introduce as much tension to an innovation project, compared to experimental or contextual learning, which can weaken the positive rhythm of team learning that is important for overall performance. In other words, by remaining grounded in more-harmonious activities focused on exploitation, teams will fail to cast a wide net on what is possible, and they will experience lower benefits from the return to reflexive learning later in the project. Therefore, we hypothesize the following:

**Hypothesis 4 (H4):** A sequence of more-dissonant (versus more-harmonious) learning activities across teamwork episodes will be associated with a greater positive rhythm of team learning for innovation project performance.

## Study 1: Testing Theory with Innovation Project Teams

### Research Setting

We tested our hypotheses by leveraging unique access to innovation project teams at a Fortune Global 500 company in the telecommunications industry, which ran an internal innovation contest. In the contest, any employees could form a team, submit an idea, and develop a project that could in some way improve or transform the organization’s products, services, or processes to enhance customers’ experience. All teams were self-organized and self-managed, and team members shared responsibility for tasks and mostly completed projects outside the formal structure of the organization (see Manz and Sims, 1987). Although most projects started out as additional sideline work for team members, management often accepted and even encouraged employees involved in the contest to spend some of their working hours on the project. Team outcomes ranged from incremental to radical innovations (Alexander and van Knippenberg, 2014), solving a wide range of problems such as improving Ethernet services in rural areas, responding to customer dissatisfaction more effectively, creating new pricing tools for salespeople, and developing stronger Internet-of-Things capabilities in their products.

At the time of data collection, the contest was being held for its eighth consecutive year. It began with 5,545 participants working on 1,122 teams, which had senior-level managers acting as project sponsors. The contest lasted for seven months and included several stages that eliminated teams from the competition over time. Teams could also voluntarily withdraw from the contest at any point. The first round occurred in month one and included all teams making progress on their projects. The quarter-finals occurred in month two and included 450 teams getting interviewed by mid-level managers about their projects. The semi-finals occurred in month four and included 50 teams delivering 20-minute pitches to senior-level managers. The grand-finals occurred in month six and included 11 teams pitching their projects to a panel of executives. Eventually, one team was selected as the overall winner, but all teams were encouraged to complete their projects by month seven, at which point they earned a blue-ribbon award from the organization. In total, 627 teams completed their projects to earn this award. Based on our observations and interactions with participants, such high participation and completion rates in the contest seemed to come from strong cultural norms that developed over several years at the company.

Indeed, the contest was a vibrant cultural phenomenon; it was the first author’s main field site during a two-year postdoctoral study. We conducted over 50 interviews with previous contest organizers, participants, and judges (i.e., senior managers) before starting data collection, in part to make sure the measures were in line with the context of the study. A dedicated five-member committee at the company managed the contest, providing branding, special events, prizes, and considerable fanfare to promote it. People at all hierarchical levels and across business units spoke highly of the contest, referring to it as an “innovation engine” for the company. We also found that emotions tied to the competition seemed intense: we observed contest artifacts such as blue-ribbon awards pinned on several employees’ cubicle walls, and many individuals expressed that they cared a great deal about not having made it to the final stage in previous years. Furthermore, although teams were most excited about the prospect of reaching the final stages to pitch their projects to top management, all teams in the competition received sponsorship from at least one executive, which we suspect was an important factor in keeping teams highly engaged in their projects. Finally, participants seemed motivated to make a positive difference at the company—a goal that their direct managers and the organizing committee often supported.

We collected multiple pieces of data about the teams, including archival data such as the business unit in which each team member worked and the stage of the competition each team reached. We invited all teams still working on their projects at the quarter-finals stage (630 teams) to complete two surveys: one in month three (T1) while they were still working on their projects (294 teams responded, a 47 percent response rate) and another in month seven (T2) soon after they had completed their projects (302 teams; 48 percent). We did not base the timing of our surveys on different stages of the competition, because these stages did not necessarily correspond to clearly defined episodes in teams (Marks, Mathieu, and Zaccaro, 2001). For example, based on our interactions with the organizing committee, we knew that many participants joined teams at the request of team captains who originally submitted projects to the competition. Therefore, during the first round, many team members had not fully engaged with their team yet, which likely limited the degree to which they had engaged in any kind of team learning. Furthermore, all but one team were eventually eliminated from the competition, meaning that competition stages did not provide meaningful target milestones for most teams in our sample.

As a result, we aimed to give teams enough time to engage in some form of collective teamwork before we surveyed them about reflexive learning, which we estimated to be the approximate midpoint of the competition (e.g., Gersick, 1988, 1989). Unfortunately, this created a time constraint on our ability to fully measure and study a complete rhythm of learning activities across three teamwork episodes, which is an issue we addressed in our follow-up study described below. Finally, we invited the senior-level managers who sponsored each project to complete a survey in month eight (T3) to rate the quality of each project (138 teams; 21.9 percent). All surveys were voluntary and confidential, and we used non-identifying codes to aggregate data across surveys before conducting analysis. We also used sampling theory suggested by Dawson (2003) to identify a cutoff participation rate for each team based on their size, yielding a total of 102 teams for analysis (16.2 percent of the initial sample).^2^ All survey items were measured with a 7-point Likert scale (1 = strongly disagree; 7 = strongly agree), and in both surveys, we asked participants to focus on the previous three months of the project when responding to items.

### Measures^3^

**Reflexive learning.** We used four items from Carter and West (1998) to measure *Reflexive learning* (T1, α_team_ = .93; T2, α_team_ = .86), which included items such as “We often reviewed our approach to getting the job done” and “We often discussed the methods used to get the job done.”

**Vicarious learning.** We used five items from Bresman’s (2010) scale to measure *Vicarious learning* **(T2, α_team_ = .87)**, which included items such as “We observed the work of others outside the team to extract lessons to be applied to the project” and “We invited people from outside the team to discuss how to avoid repeating past mistakes.”

**Contextual learning.** We also used four items from Bresman’s (2010) scale for *Contextual learning* **(T2, α_team_ = .85)**, which included items such as “We scanned the environment inside or outside the organization for market information / ideas” and “We collected technical information / ideas from individuals outside the team.”

**Team performance.** We asked the sponsor of each project to rate *Team performance* **(T3, α = .85)** based on three items used by Bresman (2010), which included the following: “This team performed well regarding the efficiency of team operations,”“This team performed well regarding the quality of its work,” and “This team performed well regarding its ability to meet project goals.” We chose these managers because they were fairly high in the organizational hierarchy and had extensive experience evaluating projects within the company. They were also well positioned to rate the performance of these specific teams based on their familiarity with the teams’ goals, strategies, and general progress during the project.

**Control variables.** We controlled for several variables known to affect team performance for innovation (Hülsheger, Anderson, and Salgado, 2009). This included *Team size*, or the number of members in each team. Because participants represented 30 different countries and eight different lines of business in the organization, teams varied in surface-level and deep-level diversity (Harrison, Price, and Bell, 1998). Therefore, we controlled for *Cultural diversity* (Jang, 2017) by constructing a Blau index for the country in which each team member worked, and we controlled for *Functional diversity* (Bantel and Jackson, 1989) by constructing a Blau index for the line of business of each team member (see Harrison and Klein, 2007). We were unable to include age and gender diversity in our study, because this information was not reported in the archival data, which precluded accurate measures for the sample. Finally, we controlled for factors unique to the contest such as *Competition stage*, which indicated how far teams progressed in the competition (stages one to four), because teams that progressed further were likely to be more motivated to complete their projects, receive higher evaluation scores, and have more positive views of their team processes and experiences (Martell, Guzzo, and Willis, 1995).

### Validity and Aggregation of Team Surveys

**Internal validity.** Our survey included 13 items for different learning activities across T1 and T2 and three items for team performance. We performed a confirmatory factor analysis to verify the validity and distinctiveness of the measures. All 16 items were modeled under their respective latent factor, and goodness-of-fit indices showed satisfactory fit for a five-factor model: χ^2^ = 132.51, df = 98 (*p* = .001), comparative fit index (CFI) = .96, incremental fit index (IFI) = .96, root mean square error of approximation (RMSEA) = .06, and standardized root mean square residual (SRMR) = .06. We tested various four-factor models that combined different learning activities according to their orientation, locus, and time of measurement, but none produced a better fit than the five-factor model. We also tested for convergent and discriminant validity, using each factor’s average variance extracted (AVE) and composite reliability (CR). The AVE values range from .59 (*Vicarious learning*) to .77 (*Reflexive learning T1*), and the CR values range from .84 to .93 (same variables). With AVE values higher than .50, CR values higher than .70, and AVE values higher than their respective maximum-shared variance (MSV), the data showed satisfactory convergent and discriminant validity (Hair et al., 2014).

**Data aggregation.** To determine whether we could aggregate data from individuals to teams, we first computed interrater agreement scores (r_wg(j)_) to test whether the variance of responses within groups was lower than the variance between groups (James, Demaree, and Wolf, 1993; LeBreton et al., 2003). Assuming a normal distribution in responses, we found that all r_wg(j)_ scores were higher than .80, indicating excellent agreement (George and James, 1993). We then calculated intraclass correlation coefficients ICC(1) and ICC(2) to assess the variance explained by team membership and the reliability of team means. We found the following results: *Reflexive learning T1* (ICC(1) = .19, ICC(2) = .50; F = 1.99, *p* < .001), *Reflexive learning T2* (.13, .38; F = 1.60, *p* < .001), *Vicarious learning* (.14, .40; F = 1.67, *p* < .001), and *Contextual learning* (.23, .55; F = 2.22, *p* < .001). While the measures for ICC(1) are satisfactory (LeBreton and Senter, 2008), those for ICC(2) did not achieve the .60 criterion recommended by some scholars (e.g., Glick, 1985). Other scholars, however, have called this criterion an “arbitrary line in the sand” that must be understood in the context of other factors (LeBreton and Senter, 2008: 835). For example, ICC(2) values are systematically higher in larger groups. Because teams in our study were relatively small, lower ICC(2) values do not necessarily indicate a lack of internal consistency (LeBreton et al., 2003; Shieh, 2016; Mathieu et al., 2020). Therefore, we felt reasonably confident in aggregating our data from individuals to teams (Bliese, 2000; Chen and Bliese, 2002).

### Analytical Strategy

We used structural equation modeling techniques to test all hypotheses (Bollen, 1989). We used moderation analysis to test Hypotheses 1 and 2, which we did by creating latent variable interaction terms composed of double-mean centered product indicators. We used mediation analysis to test Hypotheses 3 and 4. All indirect mediation effects were tested with bootstrapping techniques, which involved generating 5,000 independent samples and examining the 95 percent confidence intervals for each effect size (MacKinnon et al., 2002; Cheung and Lau, 2008).

### Results

Descriptive statistics and correlations are shown in Table 1. Unsurprisingly, *Reflexive learning T1* and *Reflexive learning T2* are significantly correlated (r = .43, *p* < .01), suggesting that teams engaging in reflexive learning in an earlier episode were more likely to engage in it in a subsequent episode. However, a considerable amount of variation between these variables remains unexplained, suggesting that other factors were important for predicting reflexive learning at T2. We also find that two control variables (*Team size* and *Functional diversity*) are correlated with both objective performance (*Competition stage*: r = .25, *p* < .05; r = .19, *p* < .05) and subjective performance (*Team performance*: r = .28, *p* < .01; r = .24, *p* < .05). These results are consistent with previous research on innovation teams (Hülsheger, Anderson, and Salgado, 2009), helping establish greater confidence in our other findings.

**Table 1.** Descriptive Statistics and Correlations for Study 1*

Variables	Mean	S.D.	1	2	3	4	5	6	7	8	9
1. Team size	6.76	.63	1								
2. Functional diversity	0.33	.27	.10	1							
3. Cultural diversity	0.21	.24	−.09	−.06	1						
4. Competition stage	2.28	.79	.25^•^	.19^•^	−.18	1					
5. Reflexive learning T1	5.57	.66	−.20^•^	.01	−.05	−.04	(.93)				
6. Vicarious learning T2	5.47	.63	−.06	−.02	−.03	.12	.36^••^	(.87)			
7. Contextual learning T2	5.21	.74	.03	.13	−.12	.28^••^	.17	.51^••^	(.86)		
8. Reflexive learning T2	5.58	.54	−.04	.11	−.09	.13	.43^••^	.54^••^	.33^••^	(.86)	
9. Team performance	6.05	.68	.28^••^	.24^•^	−.17	.24^•^	.09	.12	.15	.24^•^	(.84)

• *p* < .05; ^••^*p* < .01.

* n = 102 teams.

**Figure 1.**
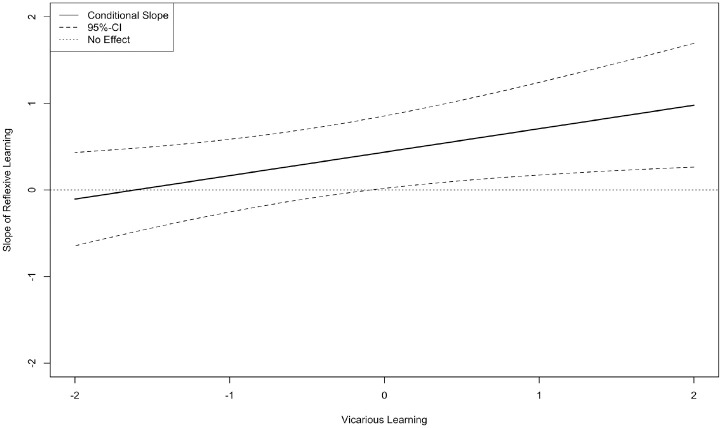
Illustration of the Slope of Reflexive Learning T2 on Team Performance at Different Values of Vicarious Learning T2 (Zone of Significance = –.13 S.D. and Above) (Study 1)

Our first hypothesis predicted that when reflexive learning occurs with other harmonious learning activities in the same teamwork episode, these activities create a positive effect on performance because they facilitate progress toward a congruent short-term goal (i.e., exploitation). We tested this by regressing *Team performance* on *Reflexive learning T2*, *Vicarious learning T2*, and their interaction. Results from this model (χ^2^_96_ = 155.51, *p* = .01, CFI = .95, TLI = .94, RMSEA = .08, SRMR = .08) show that *Reflexive learning T2* is positively associated with *Team performance* (β = .28, *p* < .05), *Vicarious learning T2* has no effect (β = –.09, *ns*), and their interaction is positively associated with *Team performance* (β = .35, *p* < .05). Using the Johnson–Neyman technique (Gardner et al., 2017), we probed this interaction by analyzing the slope of *Reflexive learning T2* on *Team performance* at several values of *Vicarious learning T2*. As Figure 1 shows, we found a region of significance above –.13 S.D. of *Vicarious learning T2*, representing 68 percent of the teams in our sample. This means that reflexive learning and vicarious learning mutually support each other for about two-thirds of the teams included in our study. H1 is thus supported.

**Figure 2.**
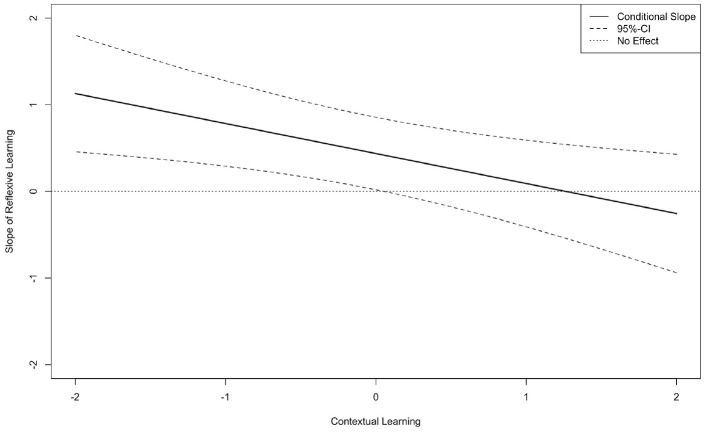
Illustration of the Slope of Reflexive Learning T2 on Team Performance at Different Values of Contextual Learning T2 (Zone of Significance = .07 S.D. and Under) (Study 1)

Our second hypothesis predicted that when reflexive learning occurs with other dissonant learning activities in the same episode, the activities will create a negative effect on performance because they facilitate progress toward conflicting short-term goals (i.e., exploration and exploitation). We tested this by regressing *Team performance* on *Reflexive learning T2*, *Contextual learning T2*, and their interaction. In this model (χ^2^_80_ = 120.29, *p* = .01, CFI = .96, TLI = .94, RMSEA = .07, SRMR = .07), results show that *Reflexive learning T2* is positively associated with *Team performance* (β = .27, *p* < .05), *Contextual learning T2* has no effect (β = .12, *ns*), and their interaction is negatively associated with *Team performance* (β = –.40, *p* < .01). Therefore, we find evidence that reflexive learning and contextual learning mutually hinder each other because they are dissonant in their goal pursuit. Probing this interaction, we show in Figure 2 a region of significance below .07 S.D. of *Contextual learning T2*, meaning that 51 percent of teams in our study were negatively affected by combining reflexive learning and contextual learning in the same teamwork episode. These results provide support for H2.

We also tested an additional model that regressed *Team performance* on *Vicarious learning T2*, *Contextual learning T2*, and their interaction. Results from this model (χ^2^_96_ = 132.43, *p* = .01, CFI = .96, TLI = .95, RMSEA = .06, SRMR = .07) show that *Team performance* is associated with neither *Vicarious learning T2* (β = .06, *ns*) nor *Contextual learning T2* (β = .10, *ns*) but is negatively associated with their interaction (β = –.27, *p* < .05). Therefore, although we did not specifically hypothesize this effect, it lends further support for our theory that combining dissonant learning activities in the same teamwork episode creates a negative effect on performance. Furthermore, we found that *Reflexive learning T2* is the only learning activity that is directly related to *Team performance*, suggesting it is indeed the tonal activity for these innovation project teams. We further validated this claim in Study 2, which more carefully measures all types of learning activities across three teamwork episodes.

Our third hypothesis predicted that two separate episodes of reflexive learning will be mediated by other learning activities to create a positive rhythm of team learning over time. We tested this hypothesis by building two mediation models: one with *Vicarious learning T2* mediating the relationship between *Reflexive learning T1* and *Reflexive learning T2* (χ^2^_97_ = 134.09, *p* < .05, CFI = .97, TLI = .96, RMSEA = .06, SRMR = .06) and another with *Contextual learning T2* mediating this relationship (χ^2^_83_ = 122.72, *p* < .05, CFI = .96, TLI = .95, RMSEA = .07, SRMR = .07). In each model, we allowed all variables to freely predict team performance. We applied metric invariance for the two measures of reflexive learning (Bliese and Ployhart, 2002). In the first model, results show that *Reflexive learning T1* is positively associated with *Vicarious learning T2* (β = .40, *p* < .001), which is positively associated with *Reflexive learning T2* (β = .43, *p* < .001). We also find that the indirect effect of *Reflexive learning T1* is positive and significant (β = .17, LLCI = .06, ULCI = .30). In the second model, we find that *Reflexive learning T1* is only weakly associated with *Contextual learning T2* (β = .19, *p* = .08) and that *Contextual learning T2* is positively associated with *Reflexive learning T2* (β = .22, *p* < .05). The indirect effect of *Reflexive learning T1* is also positive and significant (β = .04, LLCI = .001, ULCI = .14). Although these results are weak, they suggest that a positive rhythm can be achieved between both harmonious and dissonant learning activities over time. H3 is thus moderately supported.

**Figure 3.**
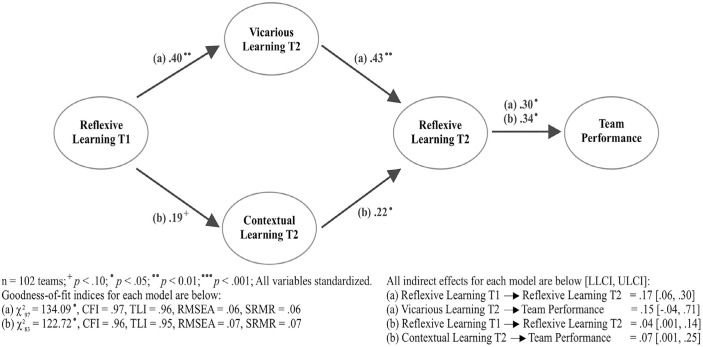
Main Results for a Positive Rhythm of Team Learning on Performance (Study 1)

Next, we assessed Hypothesis 4, which predicted that a sequence of dissonant learning activities will be associated with a more positive rhythm of team learning for innovation performance, compared to a sequence of harmonious learning activities. Results show that for both pathways, *Reflexive learning T2* is the only direct predictor of team performance (β = .34, *p* < .05; β = .30, *p* < .05). Furthermore, the indirect effect of *Vicarious learning T2* is weakly associated with *Team performance* via *Reflexive learning T2* (β = .15, LLCI = –.04, ULCI = .71), while the indirect effect of *Contextual learning T2* is positive and significant (β = .07, LLCI = .001, ULCI = .25). These results indicate that combining dissonant learning activities across teamwork episodes provides a more consistent positive effect on overall performance, compared to combining more-harmonious learning activities over time, thus providing support for H4. Figure 3 summarizes the main results of Study 1, with more-detailed results shown in Table 2.

**Table 2.** Results for a Positive Rhythm of Team Learning (H3 and H4; Study 1)*

	Vicarious Learning T2	Contextual Learning T2	Reflexive Learning T2		Team Performance	
**Direct effects**						
Reflexive learning T1	.40^••^	.19^+^	.35^••^	.48^••^	−.08	−.09
Vicarious learning T2			.43^••^		−.04	
Contextual learning T2				.22^•^		.08
Reflexive learning T2					.34^•^	.30^•^
**Indirect effects**						
Reflexive learning T1			.17^•^	.04^•^		
Vicarious learning T2					.15^+^	
Contextual learning T2						.07^•^
R^2^	.16	.04	.42	.32	.09	.09
Adj. R^2^	.15	.03	.41	.31	.06	.06

+ *p* < .10; ^•^*p* < .05; ^••^*p* < .01.

* n = 102 teams. All regression coefficients are based on standardized variables with mean = 0 and S.D. = 1. Goodness-of-fit indices for each structural equation model:

Vicarious learning T2 : χ^2^_97_ = 134.09^•^, *p* < .05 CFI = .97, TFI = .96, RMSEA = .06, SRMR = .06.

Contextual learning T2 : χ^2^_83_ = 122.72^•^, *p* < .05, CFI = .96, TFI = .95, RMSEA = .07, SRMR = .07.

### Study 1 Discussion

Study 1 offers support for our hypotheses and overarching theoretical framework, but it also has shortcomings that limit the strength of our conclusions. First, we collected longitudinal data for reflexive learning but not for other types of team learning, which prevented us from testing alternative combinations of learning activities that could have falsified our proposed theory of harmony and rhythm for innovation teams. Second, our surveys covered fairly large windows of time, which may have included multiple teamwork episodes and thus prevented us from measuring how specific team activities could have helped teams accomplish more-specific short-term goals. Third, by collecting data at only two points in time, we could not thoroughly test our theory on the rhythm of team learning activities that involves three separate episodes of team learning linked in a reciprocal cause-and-effect relationship over time (Marks, Mathieu, and Zaccaro, 2001; Ilgen et al., 2005). Finally, we did not measure experimental learning in this study, which limited our ability to run additional tests related to harmonious versus dissonant learning activities within and across episodes. To address these issues, we conducted a follow-up study with MBA teams completing a six-week class innovation project.

We also identified a theoretical shortcoming related to the successful combination of dissonant learning activities over time. Notably, our results showed that when reflexive learning and contextual learning occurred across teamwork episodes, they combined to produce an overall positive effect on performance, but the evidence for the relationship between *Reflexive learning T1* and *Contextual learning T2* was weak (*p* = .08). An early episode of reflexive learning did not seem to reliably predict a subsequent episode of contextual learning, which could have been related to the general challenges of trying to balance both exploration and exploitation in innovation projects (Edmondson, 2002; Wong, 2004; Alexander and van Knippenberg, 2014). Therefore, in addition to seeking a more stringent empirical test of our hypotheses in a second study, we also sought to elaborate our theory by identifying an intervening variable that can facilitate a stronger positive rhythm of learning activities across teamwork episodes (MacKinnon et al., 2002; Mathieu and Taylor, 2006).

## Study 2: Replicating and Elaborating Theory with MBA Teams

### Enabling Positive Rhythm Across Teamwork Episodes

At this point, our theory provides a framework to better understand how teams can arrange various activities, which can be in tension with each other, over time to achieve high levels of performance. However, what we have overlooked thus far is that these processes naturally require coordination to unfold effectively (Faraj and Sproull, 2000; Cannon-Bowers and Salas, 2001; Reagans, Argote, and Brooks, 2005). High-quality coordination, which helps teams integrate interdependent tasks to reach a common goal, relies on three conditions (Okhuysen and Bechky, 2009). First, members of well-coordinated teams display accountability, meaning they take responsibility for accomplishing tasks during a project. Second, they have predictability in their work, based on understanding both the structure of tasks and subtasks for a project and the sequence by which these tasks should be performed. Finally, these teams have a shared understanding of project goals and the strategies needed to accomplish these goals, enabling them to more clearly identify how team members fit into the overall plan.

To push our theory further, we consider coordination quality in the context of team learning, noting the connection between reflexive learning and the three conditions needed to promote high-quality coordination in teams. First, the process of identifying team members’ roles and responsibilities fosters more accountability that directs attention and guides subsequent activities (Ericksen and Dyer, 2004; Hackman and Wageman, 2005; Mathieu and Rapp, 2009; Kennedy and McComb, 2014; Mayo, 2022). Second, developing strategies and assigning tasks to team members can establish stronger shared mental models for how the project should unfold (Liang, Moreland, and Argote, 1995; Lewis, Lange, and Gillis, 2005; Ren and Argote, 2011), thus providing predictability. Finally, sharing diverse perspectives and discussing team processes can promote a stronger shared understanding of goals, tasks, and responsibilities (Salas et al., 1995; Hinsz, Tindale, and Vollrath, 1997; Gurtner et al., 2007; Ren and Argote, 2011). Therefore, coordination quality can be understood as a cognitive state that naturally emerges from team members engaging in the process of reflexive learning (Liang, Moreland, and Argote, 1995; Hoegl and Gemuenden, 2001; Lewis, 2003; He, Butler, and King, 2007; Harvey, Leblanc, and Cronin, 2019). In short, the outputs from reflexive learning provide the necessary inputs for high-quality coordination (Stout et al., 1999; Janicik and Bartel, 2003; Lewis et al., 2007), suggesting that these activities are tightly linked in an iterative cause-and-effect relationship over time (Marks, Mathieu, and Zaccaro, 2001; Ilgen et al., 2005).

The connection between reflexive learning and coordination quality has parallels in music theory, because the tonal note of a musical arrangement also helps establish a selection of notes within an octave and describes their sequence and how they relate to the original tonal note (Powers, 2001). For example, in Western music, there are seven possible tonal notes, each corresponding to a different note on the major scale and each having a distinct sound and character. Thus, when a musical arrangement begins with a tonal note, it helps define what other notes can be played to create the unique character, structure of tension and resolution, and feeling of predictability in a musical arrangement (Meyer, 1989). Similarly in team learning, the tonal activity (reflexive learning) directly builds a coordinative capacity that allows teams to move sequentially to other activities with a sense of accountability, predictability, and shared understanding.

We thus suggest that coordination quality plays an important role in facilitating the connection between dissonant learning activities such as exploration and exploitation during an innovation project. If teams engage only in reflexive learning, they might focus primarily on promoting efficiency and quality of outcomes, failing to recognize the need to obtain novel insights and additional knowledge through exploration. Generally, it is difficult for team members to engage effectively in extensive exploration without a consensus on what knowledge is needed (Marrone, Tesluk, and Carson, 2007). Therefore, coordination quality likely serves as a necessary complement to reflexive learning, helping teams recognize when and how to expand their knowledge and search for alternative strategies (Mayo, 2022). When reflexive learning fosters higher-quality coordination, teams can generate more-divergent ideas and search for alternative strategies to reach longer-term goals while also avoiding the pitfalls that can harm team cohesion during exploration (e.g., Wong, 2004). As a result, we hypothesize that coordination quality plays an essential role in teamwork arrangements for innovation, mediating the relationship between reflexive learning and other, more-exploratory types of learning, thus promoting a positive rhythm of team learning over time.

**Hypothesis 5 (H5):** Coordination quality promotes a positive rhythm of learning activities over time, such that it mediates the relationship between an early episode of reflexive learning and a subsequent episode of learning activities.

### Research Setting

To test our elaborated theory on the dynamics of team learning, we collected data on MBA teams participating in a required class on organizational design, from full-time and part-time MBA students at an elite North American university. During the class, teams needed to develop a novel solution for an organization that was dealing with some type of organizational challenge or struggle. Projects focused on a broad range of issues such as declining sales numbers, poor collaboration between business units, and difficulty retaining star employees. To address the issues, teams needed to leverage their knowledge of diverse topics such as organizational design, decision-making rights, and incentive structures. Teams were encouraged to be creative in their proposed solution because they would be evaluated based on the novelty and usefulness of their ideas. Projects took place over a six-week time frame, and teams were randomly created by the MBA program and varied in size from three to five members.

The project consisted of three clearly defined stages: identifying the problem, generating ideas, and implementing ideas in a final presentation. Each stage took two weeks to complete and concluded with an assignment summarizing the teams’ work. The first assignment required students to turn in a “problem statement” focusing on the underlying factors related to the challenge selected. The second assignment consisted of a “solution draft,” which presented a rough overview of the changes they would make in the organization to solve the problem. The last assignment was a “final presentation,” which was a recorded 15-minute PowerPoint presentation describing both the problem and solution. We collected survey data at the time of submission for each assignment, creating well-defined teamwork episodes with clear short-term goals that are consistent with the innovation process (Anderson, Potočnik, and Zhou, 2014; Perry-Smith and Mannucci, 2017; Cromwell, Amabile, and Harvey, 2018).

We invited 207 students across six sections to participate in the study over two semesters. We presented the study as an opportunity to learn more about teamwork, and participating teams were offered a team report summarizing the study results. To ensure the privacy of participants and prevent possible bias with the grading process, all data were collected and anonymized by the second author, who was affiliated with a different institution and never had contact with any of the students. Furthermore, we did not ask for identifiable information in surveys and asked participants to indicate only their team name. These names were then replaced with a randomized Team ID before data were shared back with the other authors and after the final grades were submitted to conclude the course. As a result, no author ever had full access to both the survey responses and grades from class. Out of a total of 207 students, 192 participated at T1 (93 percent), 196 at T2 (95 percent), and 201 at T3 (97 percent). Overall, 61 teams met our cutoff participation rate (Dawson, 2003), representing 100 percent of the teams in our sample.

### Measures

Given that this study aimed to replicate and elaborate findings from Study 1, we used the same measures for reflexive learning, vicarious learning, and contextual learning, but we modified the items to better fit the context of MBA project teams. We also added a new measure for experimental learning to capture exploration activities that took place inside the team, and we used a measure of coordination quality to test Hypothesis 5. Finally, to fully investigate the dynamics of team learning, we measured all types of learning activities three times at the end of each episode through a 7-point Likert scale (1 = strongly disagree; 7 = strongly agree). For all measures, we asked participants to focus on only the previous two weeks of the project when responding to items. Altogether, this approach allowed us to test a broader range of teamwork arrangements based on different learning activities occurring within and across episodes to produce different effects on overall performance.

**Team learning activities.** Reflexive learning included four items from Carter and West (1998) that were not modified for this study (T1αteam = .88; T2 αteam = .84; T3 αteam = .84). Vicarious learning included the first three items from Bresman (2010) because they best fit the context of MBA project teams (T1αteam = .85; T2 αteam = .90; T3 αteam = .88). For example, the item “We invited people from outside the team to discuss how to avoid repeating past mistakes” was modified to “We invited people from outside the team to discuss how to do well on our project.” Contextual learning included four items from Bresman (2010) that were also modified for this study (T1αteam = .88; T2 αteam = .86; T3 αteam = .86). For example, the item “We found out what competing firms or teams are doing on similar projects” was modified to “We found out what other teams are working on for this project.”

Finally, we were not aware of any existing scales for measuring experimental learning, so we drew from team behavior measures that included experimentation-focused items (e.g., Edmondson, 1999; Gibson and Vermeulen, 2003), and we generated other items by following a valid scale-development procedure (DeVellis, 2012). This involved conducting seven cognitive interviews to assess the wording of each item (Willis, 2004). After refining the wording, we collected responses from individuals in work teams, and we used exploratory factor analysis to identify the most effective items for measuring the construct. The final items to emerge from this analysis included “We engaged in a lot of trial-and-error experiments with our ideas,”“We spent a lot of time trying out new courses of action,”“We experimented with new and creative ways for accomplishing the task,” and “We often tested new ideas” (T1αteam = .84; T2 αteam = .82; T3 αteam = .86).

**Coordination quality.** We measured coordination quality (T2, αteam = .87) based on five items from Hoegl and Gemuenden (2001). Examples of items include “The work done on subtasks for this project was closely harmonized,”“There were clear and fully comprehended goals for subtasks within our team,” and “The goals for subtasks were accepted by all team members.”

**Team performance.** Performance for team projects (T3, α = .97) was measured by inviting two professional management consultants to evaluate and score the final presentations. These consultants were external to the research team and MBA program and therefore did not know this study’s hypotheses. They watched and rated each team’s presentation by using a 10-point Likert scale (1 = strongly disagree; 10 = strongly agree). Items were adapted from Weiss, Hoegl, and Gibbert (2013) as follows: “The presentation is of high quality,”“The customer or main stakeholder would be satisfied with the quality of the presentation,” and “The presentation would require little rework.” Interrater reliability was .80, which was calculated with an intraclass correlation based on consistency between raters (Neuendorf, 2002). This rating was positively correlated with the grade that teams received for the assignment (r = .40, *p* < .01), suggesting it represented a valid measure for overall team performance.

**Control variables.** We controlled for all the variables from Study 1 that were still relevant for this context. These included *team size*, which ranged from three to five members, and *industry diversity*, which was measured by constructing a Blau index for the industry background of each team member based on their career before joining the MBA program (Harrison and Klein, 2007).

### Internal Validity of Data

We assessed the structural validity of these items with confirmatory factor analysis. When modeling all 23 items on six latent factors, we found a satisfactory fit (χ^2^_215_ = 262.47, *p* = .05; CFI = .95; TLI = .94; RMSEA = .06; SRMR = .08). We tested other models that combined latent variables based on their orientation, locus, and time, but none significantly improved model fit. Convergent and discriminant validities are also adequate with AVE values higher than .50, CR higher than .70, and all MSV lower than their related AVE (Hair et al., 2014). To test whether we could aggregate data from individuals to teams, we found that r_wg(j)_ for nearly all constructs were greater than .70, except for *Vicarious learning* (.64). Moreover, ICC(1) values ranged from .09 for *Vicarious learning* (F = 1.32, *p*≤ .01) to .32 for *Experimental learning* (F = 2.54, *p*≤ .01), and ICC(2) values ranged from .24 to .61 for the same variables. Together, these results provided support to aggregate our data to teams (Bliese, 2000; Chen and Bliese, 2002; LeBreton et al., 2003; LeBreton and Senter, 2008; Shieh, 2016; Mathieu et al., 2020).

### Analytical Strategy

Similar to what we did in Study 1, we used structural equation modeling to test all hypotheses. However, we expanded the possibilities for testing hypotheses because we collected three measures of each learning activity over time. Our analytical procedure thus consisted of multiple steps to replicate and expand our findings from Study 1. For H1 and H2, this involved testing all combinations of reflexive learning and other learning activities within episodes across time. For H4, this involved testing all possible sequences of learning activities across episodes. In this study, H5 is an elaborated version of H3 due to the addition of *Coordination quality T2* as an intervening variable between *Reflexive learning T1* and other learning activities at T2. We present the full scope of these analyses in the Online Appendix and include only the most relevant results pertaining to the validity of our theory below.

### Results

Descriptive statistics and correlations are shown in Table 3. In contrast to Study 1, *Reflexive learning T1* is not correlated to *Reflexive learning T3*, indicating that other variables can explain much more of the latter’s variance. Furthermore, many learning activities at T2 and T3 are positively correlated with *Reflexive learning T3* but not with *Reflexive learning T1*, suggesting that other factors may be needed to facilitate a positive rhythm of learning activities over time. It is also noteworthy that vicarious learning is relatively lower than other learning activities across time. We suspect that the context may have affected this result, because students likely felt more competitive pressures to outperform their peers, or they may have felt that vicarious learning could have been a form of copying or cheating. As a result, they may have been less likely to exchange information with other teams during the project compared to Study 1.

**Table 3.** Descriptive Statistics and Correlations for Study 2*

Variables	Mean	S.D.	1	2	3	4	5	6	7	8	9	10	11	12	13	14	15	16
1. Team size	3.44	.83	1															
2. Industry diversity	0.67	.09	.64^••^	1														
3. Coordination quality T2	5.42	.60	−.25	−.04	(.80)													
4. Reflexive learning T1	4.24	.80	.21	.09	.26^•^	(.88)												
5. Reflexive learning T2	5.03	.73	.01	.05	.65^••^	.44^••^	(.84)											
6. Reflexive learning T3	5.29	.61	−.06	.05	.47^••^	.17	.56^••^	(.84)										
7. Vicarious learning T1	2.63	.72	.02	.10	−.02	.29^•^	.17	.24	(.85)									
8. Vicarious learning T2	3.19	.98	.09	−.06	.21	.16	.32^•^	.32^•^	.42^••^	(.90)								
9. Vicarious learning T3	3.23	.81	−.01	−.09	.31^•^	.10	.48^••^	.47^••^	.21	.58^••^	(.88)							
10. Contextual learning T1	3.53	.96	−.03	−.02	.11	.51^••^	.29^•^	.19	.50^••^	.39^••^	.30^•^	(.88)						
11. Contextual learning T2	4.55	.88	−.03	.04	.31^•^	.06	.31^•^	.42^••^	.36^••^	.63^••^	.50^••^	.45^••^	(.86)					
12. Contextual learning T3	4.52	.84	.03	.13	.35^••^	−.12	.21	.52^••^	.30^•^	.52^••^	.54^••^	.19	.71^••^	(.86)				
13. Experimental learning T1	3.60	.77	.21	.26^•^	.16	.60^••^	.30^•^	.19	.42^••^	.18	.17	.56^••^	.10	.10	(.84)			
14. Experimental learning T2	4.52	.72	−.04	.14	.47^••^	.11	.57^••^	.55^••^	.24	.47^••^	.58^••^	.28^•^	.42^••^	.51^••^	.45^••^	(.82)		
15. Experimental learning T3	4.71	.76	.03	.21	.29^•^	.04	.41^••^	.64^••^	.09	.23	.49^••^	.10	.23	.44^••^	.37^••^	.79^••^	(.86)	
16. Team performance	5.92	1.77	−.10	−.10	.10	.07	.14	.42^••^	.10	−.13	.07	.13	.10	.16	.17	.06	.24	(.97)

• *p* < .05; ^••^*p* < .01.

* n = 61 teams.

To replicate H1 and H2, we tested three structural equation models that included different interaction variables, namely *Reflexive learning T3 × Vicarious learning T3* (χ^2^_57_ = 95.99, *p* < .001, CFI = .93, TLI = .91, RMSEA = .11, SRMR = n/a since iteration limit was reached), *Reflexive learning T3 × Contextual learning T3* (χ^2^_80_ = 101.72, *p* = .05, CFI = .97, TLI = .96, RMSEA = .07, SRMR = .07), and *Reflexive learning T3 × Experimental learning T3* (χ^2^_80_ = 121.20, *p* < .001, CFI = .94, TLI = .93, RMSEA = .09, SRMR = .10). We tested for all possible interactions and found that only the last interaction effect is significant. This model shows that *Team performance* is positively associated with *Reflexive learning T3* (β = .56, *p* < .01) but not with *Experimental learning T3*, and the relationship with their interaction is negative and significant (β = –.24, *p* < .05). We probed this interaction by using the Johnson–Neyman technique (Gardner et al., 2017), finding that *Reflexive learning T3* has a positive effect on *Team performance* at .62 S.D. and under the mean of *Experimental learning T3*, representing 77 percent of our sample. In other words, the 23 percent of teams that engaged most in reflexive learning at T3 did not benefit from greater engagement in experimental learning at T3.

We believe that external learning activities such as *Vicarious learning T3* and *Contextual learning T3* did not influence the impact of *Reflexive learning T3* because MBA teams generally did not wish to conduct additional work outside the team during the project’s final teamwork episode. Given each episode’s relatively short two-week duration, the typical competitive pressures of an MBA class, and the need to balance this project with multiple other assignments at the end of a semester, it is not surprising to find that external learning activities had a weaker interaction effect on final team performance. Teams likely focused more on developing the project through internal discussions, debates, and teamwork, and therefore, these activities would have stronger predictive effects on performance. Accordingly, when testing the combination of exploration and exploitation inside teams, we found that it indeed undermined performance, complementing our findings from Study 1 to provide further support for H2.

In Study 2, we also introduced coordination quality as an intervening variable to help explain how teams can achieve a positive rhythm of team learning across teamwork episodes to achieve higher levels of performance. To test this hypothesis, we built three models in which the following relationships were tested simultaneously: *Reflexive learning T1 → Coordination quality T2 → Vicarious learning T2/Contextual learning T2/Experimental learning T2 → Reflexive learning T3 → Team performance*. As in Study 1, each model considered all direct and indirect effects concurrently (Bollen, 1989), and we applied metric invariance for the two measures of reflexive learning (Bliese and Ployhart, 2002). Fit indices were satisfactory for each model (*Vicarious learning T2*: χ^2^(141) = 142.41, *p* = .45; CFI = .99; TLI = .99; RMSEA = .01; SRMR = .07; *Contextual learning T2*: χ^2^(159) =189.22, *p* = .05; CFI = .96; TLI = .96; RMSEA = .06; SRMR = .08; *Experimental learning T2*: χ^2^(159) = 203.08, *p* = .01; CFI = .95; TLI = .94; RMSEA = .07; SRMR = .08).

For the first model, we did not find evidence for a positive rhythm operating through *Vicarious learning T2*, but the second and third models show that *Reflexive learning T1* is positively associated with *Coordination quality T2* (β = .35, *p* < .01), which is then positively associated with both *Contextual learning T2* (β = .34, *p* < .05) and *Experimental learning T2* (β = .65, *p* < .01). Furthermore, the indirect effect of *Reflexive learning T1* on *Contextual learning T2* via *Coordination quality T2* is positive and significant (β = .12, LLCI = .01, ULCI = .40), as is the indirect effect on *Experimental learning T2* (β = .23, LLCI = .04, ULCI = .51). Finally, *Contextual learning T2* and *Experimental learning T2* are both associated with *Reflexive learning T3* (β = .33, *p* < .01; β = .41, *p* < .05), with positive indirect effects coming from *Coordination quality T2* (β = .11, LLCI = .01, ULCI = .32; β = .27, LLCI = .02, ULCI = .59). Interestingly, we found that the direct relationships between *Reflexive learning T1* and *Contextual learning T2* / *Experimental learning T2* were insignificant, suggesting that coordination quality played an important role in facilitating a positive rhythm between these dissonant learning activities over time. Furthermore, when replacing *Coordination quality T2* with *Reflexive learning T2*, we found that the model fit indices became worse, and more important, the indirect effects supporting our hypotheses became insignificant. Therefore, we found fairly strong support for H5, which elaborated H3 from the previous study.

**Figure 4.**
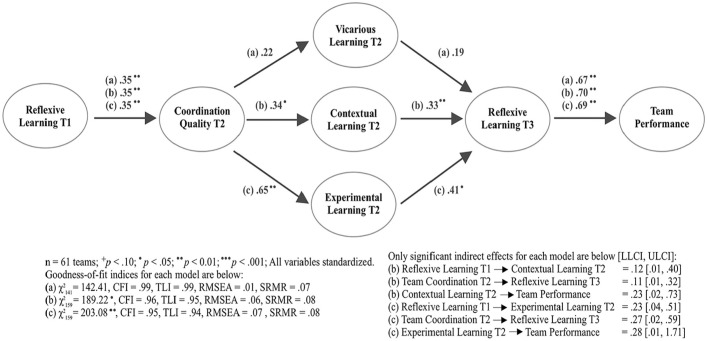
Main Results for a Modified Positive Rhythm of Team Learning on Performance (Study 2)

Finally, for H4, we found that *Reflexive learning T3* is positively associated with *Team performance* (β = .67, *p* < .01; β = .70, *p* < .01; β = .69, *p* < .01), whereas *Vicarious learning T2*, *Contextual learning T2*, and *Experimental learning T2* are not. These results again indicate that reflexive learning is likely the tonal activity for innovation projects. Note also that *Vicarious learning T2* has a negative effect on *Team performance* (β = –.26, *p* < .05), indicating that MBA teams indeed may have considered this an inappropriate activity for their projects. Furthermore, the indirect effects of *Contextual learning T2* and *Experimental learning T2* on *Team performance* via *Reflexive learning T3* are both positive and significant (β = .23, LLCI = .02, ULCI = .73; β = .28, LLCI = .01, ULCI = 1.71), while the indirect effect for *Vicarious learning T2* is not. These results provide additional support for H4. Figure 4 summarizes all results from Study 2, and the Online Appendix provides more-detailed analyses covering other possible combinations of team learning. Finally, for a robustness check, we tested all models with control variables for *Team size* and *Industry diversity* and found no significant improvements in model fit and no substantive changes to regression coefficients or z-scores for our variables.

## Discussion

In this research, we sought to show how innovation teams can achieve harmony and rhythm in team learning activities to produce high levels of performance. By integrating research on teamwork episodes (Marks, Mathieu, and Zaccaro, 2001) with insights from music theory (Albert and Bell, 2002), we generated thought trials that explicitly consider diverse conjectures on the temporal nature of team learning (see George and Jones, 2000). Specifically, we conjectured that engaging in particular activities at one point in time does not have the same effect as engaging in them at another point in time. Our research thus sheds light on how teams can best combine different team learning activities within and across teamwork episodes.

Drawing on the concept of tonality, we identified reflexive learning as the primary learning activity against which all other team learning activities are interpreted. In contrast to vicarious, contextual, or experimental learning, reflexive learning provides stability and direction when repeated over time because it allows teams to develop and update their shared mental models during a project (Schippers, Edmondson, and West, 2014). However, it must be carefully orchestrated with other learning activities for teams to reap its full benefits. Using the concepts of harmony and dissonance, we explained why some combinations of team learning can have a positive effect on performance because they help teams pursue a congruent short-term goal (e.g., exploitation), while others have a negative effect on performance because they emphasize progress toward conflicting goals (e.g., exploration and exploitation). We used the concept of rhythm to explain why dissonant learning activities can be separated across teamwork episodes to promote a positive rhythm of team learning, which facilitates a rise and resolution of tension over time that can improve overall project performance.

### Theoretical Contributions

**Contributions to team dynamics.** We extend the episodic view of team dynamics, which has argued that various activities can run sequentially or simultaneously across multiple IPO cycles (Marks, Mathieu, and Zaccaro, 2001). According to this view, team activities “may occur at any given time” because teams orchestrate multiple tasks that are not necessarily synchronized (Mathieu et al., 2020: 403; Mathieu et al., 2022). In the context of team learning, although teams can engage in any combination of reflexive, experimental, vicarious, or contextual learning at the same time, our results suggest that only one of these combinations improves performance. By explicitly accounting for the alignment or conflict between the short-term goals of different learning activities, our research provides a new lens for understanding how specific activities should be combined, or not, over time. Our overall aim is to challenge the view that all team activities can occur within and across episodes in any combination without altering performance; instead, we propose that the positioning of different types of team activities relative to one another can strongly influence outcomes. In short, different *teamwork arrangements* influence performance.

In theorizing that tonality, harmony versus dissonance, and rhythm help explain how the timing of different team activities influences innovation performance, we offer a new way to think about team dynamics as they unfold over time. We propose that team activities facilitating progress toward congruent short-term goals combine to have positive effects on performance, while activities focused on conflicting short-term goals combine to have negative effects. The latter produce a positive effect on performance only when they are separated across teamwork episodes, creating a positive rhythm of learning over time. Importantly, we theorized reflexive learning as the tonal activity for this arrangement because it provides a sense of stability and direction for a team. Departures from this activity are acceptable as long as the team returns to it at a later point in time, allowing for a predictable rise and resolution of tensions during a project (Albert and Bell, 2002). When applying this theory to other teams—especially those relying on strong routines and procedures (Gersick and Hackman, 1990)—reflexive learning may not be the tonal activity but, rather, serves as a complementary activity that increases tension because it promotes more discussion among diverse perspectives and synthesizes knowledge into new insights for a project. In this teamwork arrangement, other activities focused on coherence and predictability could be more appropriate as the tonal activity. Future research can build on our study by investigating how various other activities can create a positive rhythm of teamwork in various other contexts.

Our results also suggest new research questions related to the challenges of managing diverse team activities over time in complex arrangements. Notably, how teamwork episodes unfold in real organizations presents a crucial area for further study. For instance, the boundaries between teamwork episodes are likely to blur, as teams may engage in different activities over time without pausing to take stock of what they have accomplished to plan what they need to do next. Our results suggest that this may reflect poor coordination in a team rather than a fundamental feature of team dynamics. Future research could explore whether and how well-coordinated teams engage in distinct episodes with clearly defined short-term goals. Through superior coordination, teams may impose an episodic structure on their work that would be otherwise lacking in the team’s design. Another issue, which the lower ICCs found in our studies (notably, vicarious learning in Study 2) may reflect, is that teams may be more or less concerted in their efforts during a given episode. For example, some members engage in activities that promote exploration while others engage in activities promoting exploitation (Mayo, 2022; Venkataramani and Tang, 2023). Team size likely affects teams’ ability to do this successfully. Larger teams may be more able to divide and delegate different learning activities to subsets of members—provided they are well coordinated—but smaller teams may struggle to engage in multiple learning activities simultaneously and may suffer from a lack of harmony. These are promising avenues for future research.

Finally, future research could explore how the duration of episodes affects team dynamics, given how much they vary based on the nature of the task and project. For instance, teamwork episodes can be defined by the type of project under development and are sometimes accelerated by external pressures from managers or customers. Innovation teams typically engage in a problem-solving process that consists of multiple stages, such as defining problems, generating ideas, and implementing ideas to develop a solution (West and Farr, 1990; West, 2002; Hülsheger, Anderson, and Salgado, 2009; Anderson, Potočnik, and Zhou, 2014; Cromwell, Amabile, and Harvey, 2018). Each stage thus represents an important milestone and short-term goal for a teamwork episode, and the duration of each episode depends on how much progress is made toward the milestone. Sometimes teams need several months to find the best solution to a problem, and other times this discovery can happen through a sudden burst of insight. Overall, when a project stretches over a long period of time, breaking the project down into smaller teamwork episodes may be valuable, because short-term goals can provide incentives to team members and help them feel they are making progress toward longer-term goals (Locke and Latham, 1990). Future work could further explore the separation of team activities across teamwork episodes in different contexts and with varying degrees or forms of goal conflict.

**Contributions to team learning.** We contribute to theory on team learning by developing a dynamic theory that better accounts for the tension between different learning activities (Edmondson, 2002; Argote, Lee, and Park, 2020; Harvey et al., 2022) to explain how they combine over time to affect performance. Previous research has highlighted the trade-offs between different types of learning activities (Argote, Gruenfeld, and Naquin, 2001; Choi, 2002), offering preliminary empirical evidence suggesting that teams may perform best when rotating between them over time (Ancona, 1990; see also Gersick, 1988). This work echoes early theoretical scholarship on team effectiveness that emphasized learning cycles as the core mechanism of team development and adaptation (Kozlowski et al., 1996, 1999). In contrast, later research mostly used a static perspective to theorize about different combinations of team learning (Edmondson, Dillon, and Roloff, 2007), which led to conflicting results (e.g., Bresman, 2010 versus Wong, 2004). Our research helps resolve this contradiction by showing that it matters whether different learning activities occur within the same teamwork episode. Team members must divide their attention between different learning activities, requiring understanding of how each activity addresses the team’s goals.

Further, our study aligns with and extends previous research suggesting that teams can avoid trade-offs between exploration and exploitation by switching from one type of activity to the other (Ancona, 1990). We go beyond these findings, which suggest that teams can simply engage in one type of learning activity and then the other, and instead argue that the output from one type of learning serves as input to another, leading to cumulative effects over time that influence performance (Marks, Mathieu, and Zaccaro, 2001). We further theorize and show that reflexive learning functions as the tonal activity for innovation teams, providing stability and predictability for these projects, but interspersing more-dissonant activities between episodes of reflexive learning allows larger gains in overall performance. Resonating with music theory, our results indicate that creating and resolving a sharper tension can yield better results (Meyer, 1989). These findings also align well with the central tenets of goal-setting theory (Locke and Latham, 1990), which argues that task performance is shaped by the extent to which people engage in iterative phases of reflecting on the task, enacting new strategies, and reflecting on the task again in light of progress made. Our study confirms the structured, intentional, planned, and goal-directed nature of learning in teams (Bell, Kozlowski, and Blawath, 2012).

Finally, our study reaffirms the importance of reflexive learning for team performance (West, 1996; Edmondson, 1999; Schippers, Edmondson, and West, 2014), countering arguments that it may be overvalued (e.g., Moreland and McMinn, 2010). Some studies have found that reflexive learning weakly predicts performance in the context of other team activities (e.g., Savelsbergh, van der Heijden, and Poell, 2009; De Jong and Elfring, 2010). Our study suggests that part of the explanation may reside in the timing and quality of reflexive learning during projects and the failure to consider the rhythmic nature of the phenomenon (see George and Jones, 2000). Moreover, the value of reflexive learning is driven by how and when it combines with other activities to serve specific task goals. For example, if teams engage in only one episode of reflexive learning early in a project and do not revisit it later to update shared mental models based on new information, this may harm performance. Similarly, teams may not benefit from reflexive learning if they engage in exploration-oriented behavior at the same time. We propose that reflexive learning must be treated as an essential team skill to be honed and developed over time to have positive effects on performance. Reflexive learning is particularly valuable after teams engage in other types of learning and need to process, consolidate, and integrate new knowledge. Team coaching (Hackman and Wageman, 2005), therefore, may benefit from exploring the best times for interventions to spur reflexive learning.

**Contributions to ambidexterity.** Organizations increasingly rely on teams for innovation (Barczak, Griffin, and Kahn, 2009; Hülsheger, Anderson, and Salgado, 2009; Harvey et al., 2022), and such teams must engage in both exploration and exploitation to be successful (Taylor and Greve, 2006; Alexander and van Knippenberg, 2014). Combining these activities most effectively, with their conflicting goals, has long been an area of interest (Argote, Lee, and Park, 2020). In fact, Edmondson (2002) suggested that entire teams can focus on either exploration or exploitation (see also Wong, 2004) to allow organizations to achieve ambidexterity. Other researchers have taken a different perspective by arguing that teams can merge exploration and exploitation activities into a cumulative innovation effort (e.g., Gibson and Birkinshaw, 2004; Jansen et al., 2016; Zhang, Zhang, and Law, 2022). Our research contributes new theory to explain how teams can achieve ambidexterity through a dynamic view of team learning. Instead of pursuing exploration and exploitation simultaneously, teams can pursue them within teamwork episodes to build a predictable rhythm of cause and effect over time. The outputs of one activity are inputs to another, helping teams iterate between conflicting goals throughout a project.

Our findings thus help advance theory on ambidexterity by showing how teams can separate exploration and exploitation through different learning activities over time. Our theory, especially the concept of tonality (Albert and Bell, 2002), becomes especially useful in providing such guidance. Relatedly, prior research found that organizations can achieve ambidexterity by iterating between exploration and exploitation over time, through structural changes related to centralization and decentralization (Nickerson and Zenger, 2002; Siggelkow and Levinthal, 2003; Gulati and Puranam, 2009; Boumgarden, Nickerson, and Zenger, 2012). But such formal structural changes may not be as effective in teams because team hierarchies often emerge in informal interactions and then persist (e.g., Barker, 1993). Also, vacillation in structure can occur over very long periods of time, limiting our understanding of how the same group of individuals achieves ambidexterity (O’Reilly and Tushman, 2013). In short, prior research has not illuminated how a sequential pursuit of exploration and exploitation could be achieved at a granular level (Gupta, Smith, and Shalley, 2006; Junni et al., 2013). Our work suggests that iterative cause-and-effect relationships between activities—rather than structural changes—help achieve team ambidexterity. This insight can lead to new lines of research examining how benefits can accrue between alternating phases of exploration and exploitation over time. The result can be a richer and broader set of sequences for teams, and potentially entire organizations, to pursue (Simsek et al., 2009; Lavie, Stettner, and Tushman, 2010; Raisch and Zimmermann, 2017).

### Practical Implications

Innovation teams and the leaders who compose or guide them can gain several practical insights from our research. First, leaders should think of their team project in terms of teamwork episodes that enable the team to achieve higher performance through its learning dynamics. Leaders can steer the team toward reflexive learning in several ways. For instance, they can show humility (Leblanc, Rousseau, and Harvey, 2022) by engaging in premortems that bluntly consider potential problems at the onset of a project (Luth, Flinchbaugh, and Miles, 2022) or by acknowledging the unique, challenging nature of the project (Edmondson and Harvey, 2017). They can also cultivate psychological safety by asking probing questions (Edmondson, 1999, 2018) or by formalizing roles and responsibilities (Bresman and Zellmer-Bruhn, 2013). Some individuals may be quick to jump into search and experimentation when dealing with new, complex problems (Cromwell, Amabile, and Harvey, 2018); our research suggests leaders should focus a new team on reflexive learning to launch the most beneficial rhythm of learning.

Within teamwork episodes in innovation projects, leaders may need to remain mindful of tensions created by different types of team learning. Although the tension between exploration and exploitation is beneficial for team performance when it occurs over time, especially when resolved through a final episode of reflexive learning, it can also be detrimental to team performance when it occurs within the last teamwork episode. Leaders may want to use workflow structures so that teamwork episodes are clearly demarcated and can be managed deliberately (Kozlowski and Ilgen, 2006). Further, in the last teamwork episode, leaders may enable further reflexive learning by providing constructive feedback (Van der Vegt et al., 2010; Harvey and Green, 2022), ensuring membership stability on the team (Edmondson et al., 2003; Van der Vegt, Bunderson, and Kuipers, 2010), asking team members to work from the same location (Borgatti and Cross, 2003), or distributing the workload fairly evenly across team members (Ellis et al., 2003). Overall, we suggest that leaders view team learning as a process that unfolds over time rather than as a unique, non-repeatable experience that needs no attention after teams are launched and encouraged to engage in early learning behavior.

### Limitations of Study and Opportunities for Future Research

Although we provide several novel insights on the dynamics of team learning, our study suffers from methodological and theoretical limitations that suggest potential boundary conditions for our findings. First, we tested hypotheses with correlational data collected in field research. While the smaller sample size for Study 2 is common in team research, our results should be interpreted with caution, and replication studies would be useful.^4^ Moreover, in our hypotheses we use the term “mediation,” which conveys a causal quality to the examined relationships that cannot be inferred from correlational data (Stone-Romero and Rosopa, 2004). Although our data were longitudinal and collected from multiple sources, experimental research remains the only method capable of establishing causal relationships between team learning activities and performance. Future research can deepen our work by conducting experimental research on the dynamics of team learning in a laboratory setting using classic team exercises related to sharing and processing information (e.g., Stasser and Titus, 1985; Stasser and Stewart, 1992), or in a field experiment in which researchers manipulate specific team goals in clearly defined teamwork episodes. Such research could make additional theoretical contributions by investigating how the distribution of learning activities among team members affects the combinations within and across episodes. For example, by changing the referent “we” to “I” in our survey items (Klein and Kozlowski, 2000), we may find different distributions of team learning at different time points, with different influences on performance. In other words, such research could consider whether or when having some low-level variation in learning activities in an episode is helpful or distracting.

A second limitation of our study stems from the nature of the projects under consideration. Innovation teams require different types of learning to develop novel and useful outcomes, and they experience significant uncertainty when identifying goals of the project, choosing strategies to reach goals, and determining who they will collaborate with to accomplish the work. To navigate this uncertainty, they often follow a process that includes several stages such as defining a problem, generating and evaluating ideas, and implementing ideas to produce a final outcome (West and Farr, 1990; West, 2002; Hülsheger, Anderson, and Salgado, 2009; Anderson, Potočnik, and Zhou, 2014; Cromwell, Amabile, and Harvey, 2018). The findings from our study may be explained by the alignment between the learning activities we measured and the stage of the innovation process. For example, the earliest stage of defining the problem would naturally align with reflexive learning, when teams focus on understanding the task and identifying long-term goals. The subsequent stage of generating and evaluating ideas aligns with exploratory activities such as experimental learning and contextual learning, and the final stage of implementing ideas aligns with reflexive learning again because teams must focus on applying existing knowledge to the project to complete it by a deadline.

We would therefore expect to see teams engage in higher levels of exploration and lower levels of exploitation during an intermediate episode—and vice versa during a final episode—which is precisely what we found in our studies. Moreover, we would expect the combination of exploration and exploitation performed during a final episode to produce negative effects on performance because teams engaging in any exploration at this stage would likely be distracted from completing the task at hand. Therefore, the results found in our study may have had less to do with conflict or alignment of goals between the learning activities, as we theorized, and more to do with the alignment or conflict of particular learning activities with the timing of the innovation process. This issue could be addressed by collecting more outcome variables throughout the innovation project, to test whether the combination of different activities had positive or negative effects on short-term goals at the end of each episode. This could be done by evaluating the quality of the problem at the end of the first episode, the quality of ideas generated at the end of the second episode, and the quality of the final outcome produced at the end of the third episode.

These limitations suggest potential boundary conditions for our theory. Many teams do not face the degree of uncertainty that innovation teams experience and may have different processes, procedures, and routines for completing their work. For example, medical teams with high levels of hierarchy and routinization may find it difficult or undesirable to explore new ideas and develop new insights for their task (Edmondson, 2018; Satterstrom, Kerrissey, and DeBenigno, 2020). As a result, the teamwork arrangements theorized in this article may not be relevant because they depend on the combination of exploration and exploitation over time, or they could simply involve different activities that facilitate different team dynamics. Nevertheless, we suspect that the core insight of our study—that different combinations of activities can have positive or negative effects on performance depending on the degree to which they create harmony, dissonance, or rhythm in teamwork arrangements—will be generalizable to a broader set of teams beyond the innovation context.

Finally, given the potential influence of the Study 2 sample on vicarious learning, we recommend studying the effects of contexts on learning activities. MBA students in different cohorts rarely benefit from the extended time frame and the rich interpersonal connections required to achieve vicarious learning (Harvey, 2012; Myers, 2018). As earlier work has suggested (e.g., Darr, Argote, and Epple, 1995; Baum and Ingram, 1998), the importance of enduring relationships may enable teams to benefit from this type of learning. Future research on the role of intergroup leadership in such contexts—to counter a lack of enduring relationships—might be fruitful.

### Conclusion

The importance of innovation will only continue to grow in modern organizations. Understanding how innovation teams combine various learning activities over time to achieve high levels of performance thus has both theoretical and practical importance. Our research develops a new theoretical perspective on team dynamics to explain how teams can arrange different types of learning over time to navigate conflicting short-term project goals. By providing empirical support for the idea that the concepts of harmony and rhythm can shape learning dynamics, this study takes a small step toward illuminating some of the persistent mysteries of team dynamics, team learning, and ambidexterity. We hope that future research can continue building on these ideas to elaborate our understanding of the dynamics of team learning.

## Supplemental Material

sj-pdf-1-asq-10.1177_00018392231166635 – Supplemental material for The Dynamics of Team Learning: Harmony and Rhythm in Teamwork Arrangements for Innovation Click here for additional data file. Supplemental material, sj-pdf-1-asq-10.1177_00018392231166635 for The Dynamics of Team Learning: Harmony and Rhythm in Teamwork Arrangements for Innovation by Jean-François Harvey, Johnathan R. Cromwell, Kevin J. Johnson and Amy C. Edmondson in Administrative Science Quarterly

## Footnotes

1 We wish to emphasize that music theory is epistemologically compatible with research on team learning and therefore well suited for cross-disciplinary theory integration (Shaw et al., 2018). Like research in the team learning domain (see Harvey et al., 2022), the study of music occurs in the realms of human behavior, and it “is for the most part concerned to explain patterns and relationships on the levels of rules and strategies rather than on the levels of universal laws” (Meyer, 1989: 49).

2 We tested for possible response bias in our data by running a series of ANOVAs. We first compared our final sample to (a) the 238 teams that responded to T2 only and fit our cutoff participation rate for aggregation, and (b) the 137 teams that responded to both T1 and T2 and fit our cutoff rate, but for which we did not receive performance data from senior-level managers who had sponsored their project. In both cases, results suggested no significant mean differences on our independent variables. We also retested our hypotheses based on larger samples, finding no improvement in model fit and marginal differences in regression coefficients and their statistical significance. Rogelberg and Stanton (2007) have argued that replicating a set of findings across multiple data samples is another compelling method of demonstrating an absence of substantive response bias.

3 At the time of data collection, we were unaware of a valid scale to measure experimental learning in teams. Therefore, to capitalize on the research opportunity quickly, we primarily used theory to guide hypothesis development and survey design based on existing scales for Study 1, and we elaborated our theory and survey design in Study 2 to respond to any shortcomings.

4 We ran a regression power analysis based on our sample size and for each predicted (regressive) step in our models. Specifically, following Cohen (1988) and colleagues, we computed a post hoc achieved statistical power, based on our model sample size (α = 0.05), on Cohen’s f^2^ values and parameters in predicting each variable in a stepwise manner. An 80 percent power was achieved for each significant relationship in our different models except for the relationship between *Contextual learning T2* and *Coordination quality T2* (64 percent).
