# Supplementary material for: The Dynamics of Team Learning: Harmony and Rhythm in Teamwork Arrangements for Innovation
Source: Adm Sci Q. 2023 Apr 8;68(3):601–47. doi: 10.1177/00018392231166635 (PMC10406573; doi:10.1177/00018392231166635)
Supplement: sj-pdf-1-asq-10.1177_00018392231166635 – Supplemental material for The Dynamics of Team Learning: Harmony and Rhythm in Teamwork Arrangements for Innovation [file sj-pdf-1-asq-10.1177_00018392231166635.pdf]

ONLINE APPENDIX. Results of All Rhythms of Team Learning Using Structural Equation Modeling (Study 2)

1. Results for a Positive Rhythm of Team Learning with Reflexive Learning Serving as the Tonal Activity*

	Coordination T2	Vicarious Learning T2	Contextual Learning T2	Experimental Learning T2	Reflexive Learning T3			Team Performance		
**Direct effects**					VL T2	CL T2	EL T2	VL T2	CL T2	EL T2
Reflexive learning T1	.35••	.07	–.07	–.17	–.03	.01	.05	.10	.06	.04
Coordination quality T2		.22	.34•	.65••	.50••	.43••	.28	–.27	–.26+	–.19
Vicarious learning T2					.19			–.26•		
Contextual learning T2						.33••			–.25+	
Experimental learning T2							.41•			–.25
Reflexive learning T3								.67••	.70••	.69••
**Indirect effects**										
Reflexive learning T1		.08	.12•	.23•	.21••	.17•	.12	–.02	.02	.04
Coordination quality T2					.04	.11•	.27•	.31••	.30••	.22
Vicarious learning T2								.13		
Contextual learning T2									.23•	
Experimental learning T2										.28•
R^2	.13	.07	.12	.38	.32	.38	.39	.33	.32	.30
Adj. R^2	.12	.05	.10	.37	.30	.36	.37	.30	.29	.27

$^{+}$ $p \le .10$; $^{\bullet}$ $p \le .05$; $^{\bullet\bullet}$ $p \le .01$.

* n = 61 teams; All regression coefficients are based on standardized variables with mean = 0 and S.D. = 1. Goodness-of-fit indices for each sequential learning pathways:

Vicarious learning T2 : $\chi^2(141) = 142.41$, $p = .45$; CFI = .99; TLI = .99; RMSEA = .01; SRMR = .07

Contextual learning T2 : $\chi^2(159) =189.22$, $p = .05$; CFI = .96; TLI = .96; RMSEA = .06; SRMR = .08

Experimental learning T2 : $\chi^2(159) = 203.08$, $p = .01$; CFI = .95; TLI = .94; RMSEA = .07; SRMR = .08

2. Results for a Positive Rhythm of Team Learning with Vicarious Learning Serving as the Tonal Activity*

	Coordination T2	Reflexive Learning T2	Contextual Learning T2	Experimental Learning T2	Vicarious Learning T3			Team Performance		
Vicarious learning T1	–.02	.17	.31$^{\bullet}$	.20	.20	.12	.15	.08	.13	.11
Coordination quality T2		.76$^{\bullet\bullet}$	.32$^{\bullet}$	.60$^{\bullet\bullet}$	–.03	.15	–.08	–.13	.07	.07
Reflexive learning T2					.45$^{\bullet}$			.25		
Contextual learning T2						.50$^{\bullet\bullet}$			–.15	
Experimental learning T2							.66$^{\bullet\bullet}$			–.06
Vicarious learning T3								.01	.13	.09
R^2	.00	.59	.20	.39	.26	.37	.43	.04	.03	.02
Adj. R^2	.00	.58	.18	.37	.21	.34	.39	.00	.00	.00

$^{\bullet}$ $p \leq .05$; $^{\bullet\bullet}$ $p \leq .01$.
* n = 61 teams; All regression coefficients are based on standardized variables with mean = 0 and S.D. = 1.
Goodness-of-fit indices for each sequential learning pathways:
Reflexive learning T2 : $\chi^2(124) = 131.54$, $p = .31$; CFI = .99; TLI = .99; RMSEA = .03; SRMR = .07
Contextual learning T2 : $\chi^2(124) =147.52$, $p = .07$; CFI = .97; TLI = .97; RMSEA = .06; SRMR = .08
Experimental learning T2 : $\chi^2(124) = 141.35$, $p = .14$; CFI = .98; TLI = .97; RMSEA = .05; SRMR = .07

3. Results for a Positive Rhythm of Team Learning with Contextual Learning Serving as the Tonal Activity*

	Coordination T2	Reflexive Learning T2	Vicarious Learning T2	Experimental Learning T2	Contextual Learning T3			Team Performance		
Contextual learning T1	.10	.27••	.42••	.25	.25	–.02	.08	.06	.29	.16
Coordination quality T2		.71••	.20	.57••	.45•	.21	.02	–.19	.01	.06
Reflexive learning T2					–.19			.24		
Vicarious learning T2						.55••			–.48••	
Experimental learning T2							.51••			–.13
Contextual learning T3								.16	.39•	.18
R^2	.01	.62	.23	.41	.17	.38	.30	.07	.18	.05
Adj. R^2	.00	.61	.21	.38	.12	.36	.25	.00	.12	.00

• $p \leq .05$; •• $p \leq .01$.
* n = 61 teams; All regression coefficients are based on standardized variables with mean = 0 and S.D. = 1.
Goodness-of-fit indices for each sequential learning pathways:
Reflexive learning T2 : $\chi^2(159) = 171.00$, $p = .24$; CFI = .99; TLI = .98; RMSEA = .04; SRMR = .06
Vicarious learning T2 : $\chi^2(141) = 173.08$, $p = .03$; CFI = .96; TLI = .96; RMSEA = .06; SRMR = .07
Experimental learning T2 : $\chi^2(159) = 234.23$, $p = .00$; CFI = .91; TLI = .90; RMSEA = .09; SRMR = .09

4. Results for a Positive Rhythm of Team Learning with Experimental Learning Serving as the Tonal Activity*

	Coordination T2	Reflexive Learning T2	Vicarious Learning T2	Contextual Learning T2	Experimental Learning T3			Team Performance		
Experimental learning T1	.21	.20$^{\bullet}$	.10	.02	.25	.30$^{\bullet}$	.30$^{\bullet}$	.10	.14	.12
Coordination quality T2		.70$^{\bullet\bullet}$	.23	.32$^{\bullet}$	.07	.24	.22	–.15	–.01	–.03
Reflexive learning T2					.28			.15		
Vicarious learning T2						.10			–.17	
Contextual learning T2							.15			–.06
Experimental learning T3								.21	.24	.23
R^2	.04	.59	.07	.10	.23	.21	.22	.09	.11	.08
Adj. R^2	.02	.58	.04	.07	.21	.19	.20	.05	.05	.04

$^{\bullet}$ $p \leq .05$; $^{\bullet\bullet}$ $p \leq .01$.
* n = 61 teams; All regression coefficients are based on standardized variables with mean = 0 and S.D. = 1.
Goodness-of-fit indices for each sequential learning pathways:
Reflexive learning T2 : $\chi^2(159) = 211.33$, $p = .00$; CFI = .94; TLI = .93; RMSEA = .07; SRMR = .08
Vicarious learning T2 : $\chi^2(141) = 182.43$, $p = .01$; CFI = .95; TLI = .94; RMSEA = .07; SRMR = .08
Contextual learning T2 : $\chi^2(159) = 212.30$, $p = .00$; CFI = .94; TLI = .92; RMSEA = .08; SRMR = .09
